# A NETWORK ANALYSIS OF HOW COMPONENTS OF WELL-BEING DIFFER ACCORDING TO EDUCATION ATTAINMENT

**DOI:** 10.1093/geroni/igae098.4102

**Published:** 2024-12-31

**Authors:** Shuna S Khoo, Zhihao Ma, Lile Jia

**Affiliations:** National University of Singapore, Singapore, Singapore; Nanjing University, Nanjing, Jiangsu, China (People’s Republic); National University of Singapore, Singapore, Singapore

## Abstract

Singapore is on the cusp of ushering in a new wave of older adults. As one of the Four Asian Tigers, the country underwent rapid industrialization in the 1950s - 90s and buttressed its development upon a foundation of high education attainment for its citizens. This has created a disparity in the educational profile of our current (low educational attainment) and future (high educational attainment) older adults. To future-proof our aging interventions and programs, there is an urgent need to understand how different factors define and affect individuals’ well-being based on their educational attainment. We surveyed a representative community sample (N = 5,224, Mage = 52.8 years) and, employing a network analysis, mapped out the relationships among the key elements of well-being. Machine learning techniques revealed distinct network patterns between low- and high-education adults (p <.001). Low-education residents’ well-being revolves around factors relating to their present life conditions (e.g., chronic illnesses, eating habits, quality of life). By contrast, high-education residents’ well-being is centered around factors that relate to an overall sense of balance (e.g., feelings of harmony) and a future orientation (e.g., anticipated quality of life in ten years). Our findings can be used to assess well-being interventions based on how they impact the central factors for low- and high-education residents, respectively. The present methods and findings would be relevant to any fast-developing country; understanding the residents’ well-being profiles is critical to finetuning support for current older adults while anticipating future older adults’ needs regarding longevity.

